# CNSA: a data repository for archiving omics data

**DOI:** 10.1093/database/baaa055

**Published:** 2020-07-23

**Authors:** Xueqin Guo, Fengzhen Chen, Fei Gao, Ling Li, Ke Liu, Lijin You, Cong Hua, Fan Yang, Wanliang Liu, Chunhua Peng, Lina Wang, Xiaoxia Yang, Feiyu Zhou, Jiawei Tong, Jia Cai, Zhiyong Li, Bo Wan, Lei Zhang, Tao Yang, Minwen Zhang, Linlin Yang, Yawen Yang, Wenjun Zeng, Bo Wang, Xiaofeng Wei, Xun Xu

**Affiliations:** 1 China National GeneBank, Shenzhen 518120, China; 2 BGI-Shenzhen, Shenzhen 518083, China; 3 Guangdong Provincial Key Laboratory of Genome Read and Write, Shenzhen 518120, China

## Abstract

With the application and development of high-throughput sequencing technology in life and health sciences, massive multi-omics data brings the problem of efficient management and utilization. Database development and biocuration are the prerequisites for the reuse of these big data. Here, relying on China National GeneBank (CNGB), we present CNGB Sequence Archive (CNSA) for archiving omics data, including raw sequencing data and its further analyzed results which are organized into six objects, namely Project, Sample, Experiment, Run, Assembly and Variation at present. Moreover, CNSA has created a correlation model of living samples, sample information and analytical data on some projects. Both living samples and analytical data are directly correlated with the sample information. From either one, information or data of the other two can be obtained, so that all data can be traced throughout the life cycle from the living sample to the sample information to the analytical data. Complying with the data standards commonly used in the life sciences, CNSA is committed to building a comprehensive and curated data repository for storing, managing and sharing of omics data. We will continue to improve the data standards and provide free access to open-data resources for worldwide scientific communities to support academic research and the bio-industry.

Database URL: https://db.cngb.org/cnsa/.

## Introduction

In the data-intensive science era, life science research is seen as a data-driven, exploration-centered style of science. With the development of sequencing technology, the rapid increase of sequencing throughput and the dramatic drop of sequencing cost have made large-scale population genomics research, precision medicine research and biodiversity research possible. For instance, the UK’s 100 000 Genomes Project [1], the International Cancer Genome Consortium (ICGC) [2], the Cancer Genome Atlas (TCGA) [3], the China Kadoorie Biobank (CKB) [4] and Earth BioGenome Project (EBP) [5] have been announced or completed in the past decades. However, it poses great challenges in big data deposition, integration and sharing.

Many organizations in the world have made great efforts for archiving and sharing of omics data. The International Nucleotide Sequence Database Collaboration (INSDC) [6] represents one of the most celebrated global initiatives in data and associated metadata sharing, which operates between DNA Data Bank of Japan (DDBJ) [7], the European Molecular Biology Laboratory-European Bioinformatics Institute (EMBL-EBI) [8] and the National Center for Biotechnology Information (NCBI) [9]. In order to facilitate exchange of information on genomic samples and their derived data, the Global Genome Biodiversity Network (GGBN) Data Standard [10] is intended to provide a platform to promote the efficient sharing and usage of genomic sample material and associated specimen information in a consistent way. Global Alliance for Genomics and Health (GA4GH) [11], an international, nonprofit alliance, already brings together more than 500 leading organizations to accelerate the progress in genomic research and human health. In addition, DataCite [12] has developed tools and methods to make data more accessible and more useful. In China, many scientific institutions have also made great efforts and established multiple omics database systems such as the National Genomics Data Center (NGDC) [13], Bio-Med Big Data Center (BMDC: https://www.biosino.org/bmdc/index) and the National Center for Protein Science•Shanghai (NCPSS: http://www.sibcb-ncpss.org/index.action).

The China National GeneBank (CNGB) [14] was established in January 2011, which is committed to supporting public welfare, life science research, innovation and industry incubation, through effective bioresource conservation, digitalization and utilization. Based on this concept and relying on the CNGB, the China National GeneBank DataBase (CNGBdb: https://db.cngb.org/) has been built as a unified platform built for biological big data sharing and application services to the research community. Based on the big data and cloud computing technologies, it provides data services such as archive, analysis, knowledge search, management authorization and visualization. CNSA is the data archiving system of CNGBdb and is built for archiving omics data including not only raw sequencing data but also further analyzed results. At present, CNSA follows the data standards and structures of INSDC, DataCite, GA4GH and GGBN for data compatibility and provides global users with data archival and sharing services of omics data such as data submission, data storage, data retrieval and data reference. All archived public data is freely available to worldwide scientific communities.

## Methods

### System structure

Based on the Django framework which is a high-level Python Web framework for web development and maintenance (https://www.djangoproject.com/), CNSA is developed in Python. In order to provide more stable and fast services, the CNSA server is built on the Centos-7 operating system with the following six servers: NGINX for providing static resource access, uWSGI for deploying services, PostgreSQL and MongoDB for supporting metadata storage, FTP server for uploading and storing data files and Elasticsearch for data retrieval. In addition, Redis-based caching system is used to help improve data verification speed.

### Data security

The CNGB has passed the three-level review of information security level protection and the protection capability review of trusted cloud service. Relying on the CNGB, CNSA adopts corresponding security technologies in user access, data room, firewall, application architecture, database and data storage. CNSA uses https to encrypt requests of user access to prevent stealing and tampering during data transmission. Django ORM is used to avoid SQL injection, and fields retrieved from the database are filtered before being displayed to prevent XSS attacks. All information is submitted in Post mode, and Django’s CsrfViewMiddleware is used to prevent CSRF attacks. The firewall security technology of the CNGB data room can ensure the legality of data access. Moreover, CNSA adopts high-performance distributed object storage for data archiving. The database is backed up daily and can be restored quickly.

## Results

### Data objects and structure

**Figure 1:**
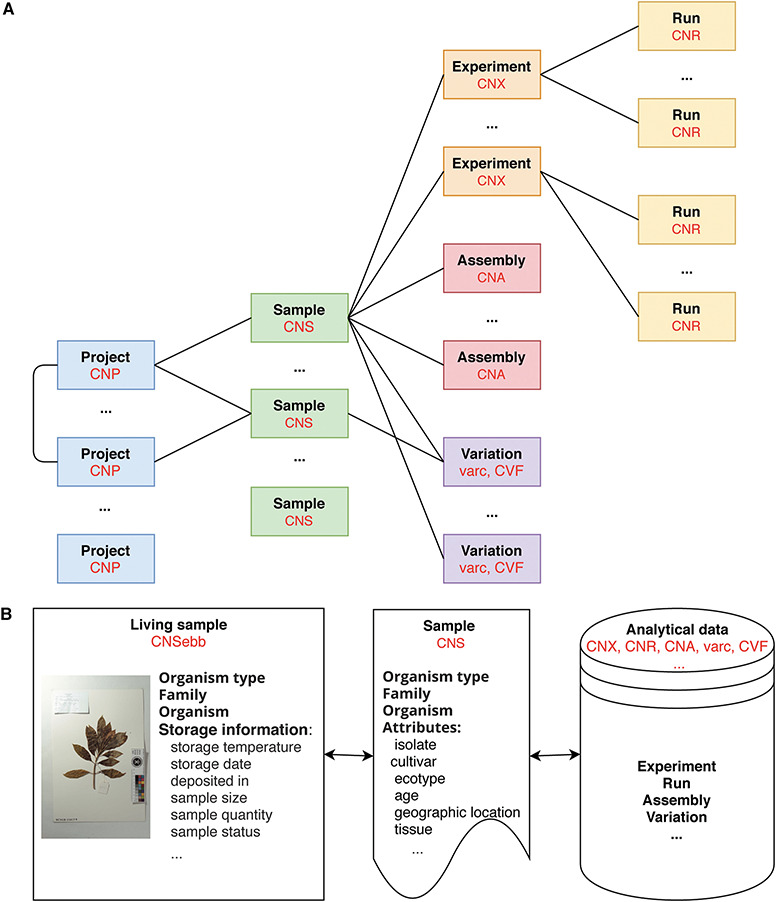
Data model in CNSA **A.** At present, CNSA has six data objects, and the corresponding prefixes of accession numbers are marked in red. **B.** Correlation model for Ruili Botanical Garden project.

At present, CNSA follows the data standards and structures of INSDC, DataCite, GA4GH and GGBN to ensure data compatibility. All data are organized into six objects, i.e. Project, Sample, Experiment, Run, Assembly and Variation. The definitions of data objects and main fields describing the data objects are listed in Table 1. As is illustrated in Figure 1A, projects and samples can be submitted independently. A project can be associated with one or more projects, and a sample can be associated with one or more experiments, assemblies or variations. An experiment can be associated with one or more runs. Moreover, each data is assigned a corresponding accession number (for example, project: CNP0000126, sample: CNS0020690, experiment: CNX0023584, run: CNR0028196, assembly: CNA0000829) that can be used for reference and search. Also note that projects and samples are not directly related, and they are not related until experiments, assemblies or variations are associated with projects and samples, respectively.

**Table 1:** Definitions and main fields of data objects

Data object	Definition	Main fields
Project	An overall description of a single research initiative	Project name, project title, public description, sample scope, data type, submitter, funding information, publication
Sample	A description of biological source material	Sample type, sample name, organism, taxonomy ID, collection, isolate, tissue, location, phenotype, disease
Experiment	A description of sample-specific sequencing library, instrument and sequencing methods	File type, sequencing platform, library strategy, library source, library layout
Run	A description of the sequencing data files that belong to the related experiment	File name, MD5 value
Assembly	A collection of genomic sequences that are used to represent the genome of an organism.	Molecule type, coverage, sequencing technology, assembly method
Variation	Genome variations of any species	Variation type, position, variation, detection method, clinical significance, phenotype, condition

#### 

It is worth mentioning that CNSA has created a correlation model of living samples, sample and analytical data on some projects such as the Ruili Botanical Garden project (https://db.cngb.org/search/project/CNPhis0000538) [15] and Culturable Genome Reference (https://db.cngb.org/search/project/CNP0000126/) [16]. Figure 1B illustrates the interrelationship of the living samples, sample and analytical data for the Ruili Botanical Garden project. CNSebb is the prefix of the accession number of the living samples which can be applied to the E-BioBank (EBB: https://db.cngb.org/ebb/), a shared platform for sample resources in the CNGB. Take a sample (CNSebb2000255) from the Ruili Botanical Garden project as an example, CNSebb2000255 is the living sample accession. On the detail page of this living sample (https://db.cngb.org/search/sample/CNSebb2000255/), the associated sample information (CNShis0046812) and its related analysis data (experiment/run: CNXhis0119000/CNRhis0123700) can be retrieved, so that all data can be traced throughout the life cycle from the living sample to the sample information to the analytical data.

### Data submission and curation

**Figure 2:**
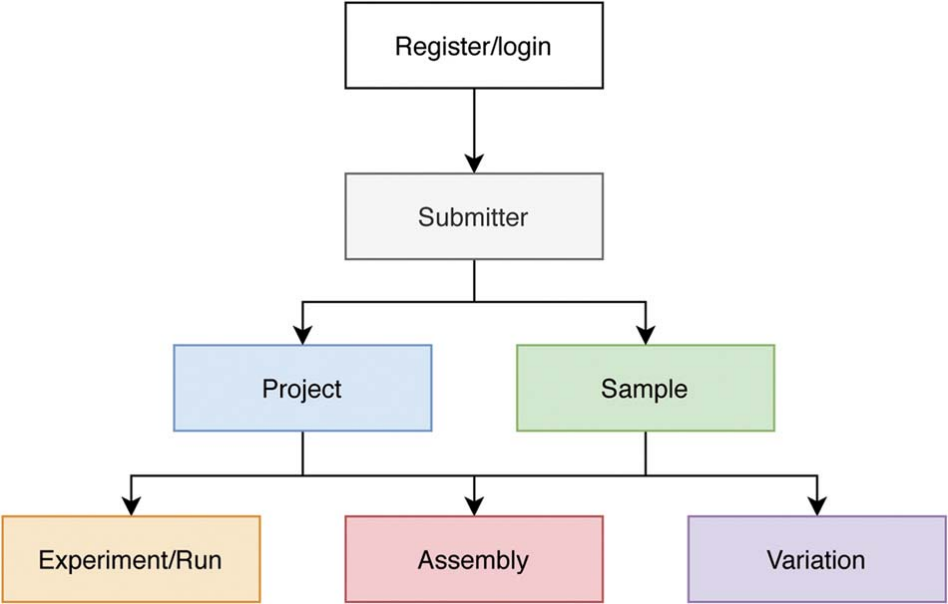
Process of data submission to CNSA.

Users need to register, login and fill in the submitter’s information before creating a submission. Generally, the order of data submission is project, sample and related experiments, runs, assemblies and variations (Figure 2). The data to be submitted includes metadata and data files. At its most basic level, metadata is ‘data about data’. Here metadata is data that describes a data object, such as the attributes listed in the ‘Main Fields’ column of Table 1. To collect metadata, CNSA provides a user-friendly and easy-to-use submission process that supports Chinese and English bilingual interfaces. It is worth mentioning that CNSA supports batch submission of samples, experiments, runs, assemblies and variations. Therefore, users can fill in a batch submission template and submit a batch of data in one submission process. Compared to a single submission, batch submission greatly improves the efficiency of data submission. Users can choose single submission or batch submission according to the number of entries. To simplify the submission of data files, CNSA supports data files to be uploaded via FTP. Moreover, in order to ensure the integrity of the submitted data, users need to submit the corresponding MD5 checksums of data files while submitting the metadata. The system will also automatically check the standardization of some field information submitted by users, such as format and character limit. It verifies the MD5 checksums of the data files and gives corresponding prompts if there are errors. After the data is submitted successfully, each piece of data will be assigned an accession number, and each project will be assigned a DOI which is a persistent identifier.

#### 

In addition, like INSDC members, there are two data management manners for projects, public and controlled. The data submitter can choose a data management manner of the project. All metadata and data files associated with the project set to be public will be public on the release date set by the submitter. The public data will be open to the world, and users can access or use it freely without logging in or registering. However, the metadata associated with the project set to be controlled will be public on the metadata release date set by the submitter, and the data files will be under controlled access. Other registered users can submit an application to the CNGB Data Access (CDA: https://db.cngb.org/data_access/) to apply for access to controlled data. Data applicants can use the controlled data only after the data access application has been reviewed and approved. After the project is successfully submitted, it will undergo materials review, such as review of materials related to ethics or human genetic resources. All submitted data must pass the material review before data biocuration. It can only be released or controlled after being approved by biocurators. In addition to online check of standardization of some field information, the metadata will also be manually reviewed to ensure its completeness, relevance and correctness. In order to increase the reusability of data, CNSA only accepts data files in commonly used formats, such as FASTQ, BAM, FASTA, VCF. Moreover, quality control such as checking the correctness of the formats and statistic the data quality using fastp [17] is performed on raw sequencing data files in FASTQ format.

**Figure 3:**
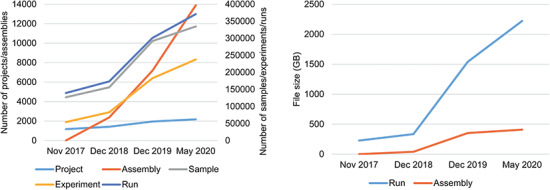
Data statistics of CNSA **A.** Numbers of Projects, Samples, Assemblies, Experiments and runs in CNSA. **B.** File sizes of Runs and Assemblies in CNSA. All statistics are based on data submitted from November 2017 to May 2020.

**Table 2:** Summary of sequence types and amount of several sequence archive databases

Database	Sequence types	Amount
INSDC	Next-generation reads, capillary reads, annotated sequences	7.2 trillion bases^a^
TCGA	Genomic, epigenomic, transcriptomic and proteomic sequence reads for tumor and normal samples	1.4 petabyte^b^
GSA	Raw sequence reads of omics	2.3 petabyte^c^
CNSA	Raw sequence reads of omics and assemblies	2.6 petabyte^d^

^a^Based on the data statistics of release 119: https://www.ddbj.nig.ac.jp/stats/release-e.html#data_category

^b^Based on the data statistics of May 24, 2020: https://portal.gdc.cancer.gov/repository?filters=%7B%22op%22%3A%22and%22%2C%22content%22%3A%5B%7B%22op%22%3A%22in%22%2C%22content%22%3A%7B%22field%22%3A%22files.data_category%22%2C%22value%22%3A%5B%22sequencing%20reads%22%5D%7D%7D%5D%7D&searchTableTab=files

^c^Based on the statistics of May 24, 2020: https://bigd.big.ac.cn/gsa/

^d^Based on the statistics of May 24, 2020: https://db.cngb.org/cnsa/statistic/

### Data archive and statistics

Currently, CNSA archives omics data from around the world, including six objects (Project, Sample, Experiment, Run, Assembly, Variation). The data is made public or controlled based on the submitter’s settings. To ensure data security, CNSA adopts high-performance distributed object storage for data archiving, and double data backup on physically independent disks.

As of May 24, 2020, CNSA has archived a total of 2177 projects, 334 824 samples, 237 859 experiments, 371 066 runs, and 13 890 assemblies for 3079 species (Figure 3A), submitted by 470 submitters from 108 institutions. The total amount of archived run files and assembly files have reached 2631 TB (Figure 3B). Moreover, CNSA has supported 124 articles published in 82 journals.

#### 

We summarized the type and amount of sequence data archived in several sequence archive databases such as INSDC, TCGA and Genome Sequence Archive (GSA) [18] (Table 2), which will be helpful for the users when selecting specific databases for bioinformatics research.

### Data retrieval and reference

As mentioned in the submission process, each piece of data will be assigned an accession number, and each project will be assigned a DOI which is a persistent identifier. All public data can be searched in CNSA by accession numbers of Project, Sample, Experiment, Run, Assembly or any other combination of keywords. For example, if users are interested in collecting data for a genome-wide association study (GWAS) on breast cancer in women, they can directly enter any keyword in the search input box on the CNSA homepage (https://db.cngb.org/cnsa/), such as GWAS. Since CNSA and CNGBdb share the same search engine, the search page will automatically jump to CNGBdb. Users can also use CNGBdb’s advanced search (https://db.cngb.org/search/advanced/all/). First, select a database and then add multiple search keywords to filter, such as (GWAS) AND (breast cancer) AND (young women), users can also filter the results by checking some filter conditions on the left side of the page. All public data files can be freely accessed through the FTP site (ftp://ftp.cngb.org/). Moreover, both the data accession number and DOI assigned by CNSA can be used to reference the submitted data to support the publication of scientific research results.

### Conclusions and perspectives

In conclusion, CNSA is a data repository for archiving omics data, including raw sequencing data and its analysis result. Currently, online submissions of projects, samples, experiments, runs, assemblies and variations are available. Moreover, compared with similar databases, an advantage worth mentioning is that CNSA has created a correlation model of living samples, sample information and analytical data on some projects. From now on, CNSA will practice the correlation model on more projects to make all data can be traced throughout the life cycle from the living sample to the sample information to the analytical data and promote the scientific and rational use of biological living samples. All public data resources of CNSA are freely worldwide scientific communities. In compliance with data standards commonly used in the life sciences, CNSA is committed to building a comprehensive and curated data repository for the storage, management and sharing of omics data, and improving the data standards to alleviate the growing management pressure of biological big data and support academic research and the bio-industry.

In order to promote the sharing and exchange of information, technology and resources of life science and facilitate the rational and efficient use of life resources, CNSA will continue to upgrade and expand. The infrastructure will be upgraded to improve the efficiency of the system and user experience. In addition, new data types associated with the omics data such as sequence, protein, metabolism, expression, clinic and image will be gradually added to the database to enrich our database and meet the needs of more users in the future.

## Notes

X.G. and F.C. contributed equally to this work.
